# The Twenty-Third Long Fox Memorial Lecture: Some Aspects of Heart Disease in Childhood

**Published:** 1934

**Authors:** F. J. Poynton

**Affiliations:** Consulting Physician to University College Hospital and to the Hospital for Sick Children, Great Ormond Street


### The Bristol Medico-Chirurgical Journal

"*Scire est nescire, nisi id me*
*Scire alius sciret.*"

WINTER, 1934.

### THE TWENTY-THIRD LONG FOX MEMORIAL LECTURE:

DELIVERED IN THE UNIVERSITY OF BRISTOL ON TUESDAY, JULY 3RD, 1934.

THE VICE-CHANCELLOR (Dr. T. LOVEDAY, M.A., LL.D.) *in the Chair.*

BY

F. J. POYNTON, M.D., F.R.C.P.,

*Consulting Physician to University College Hospital and to the Hospital for Sick Children, Great Ormond Street.*

ON

### SOME ASPECTS OF HEART DISEASE IN CHILDHOOD.

It gives me unusual pleasure to have been honoured by the invitation to give the Long Fox Lecture, and to follow in the paths of those distinguished men who have already held this post.

Once more I see myself a lad of seventeen walking from St. Philip's Station across Bristol Bridge and the Drawbridge, as it was in those days, then upward

R

past the Merchant Tailors' School to the University College. There was no University with its commanding tower, but there were great teachers in that college —Professor Lloyd-Morgan, Sir William Ramsay, Dr. Young and Professor Leipner—who taught me the love of scientific knowledge and the methods of scientific inquiry. After many years I returned here as an External Examiner in Medicine, and to-day I am acting as your Lecturer.

Needless to say, I have thought much as to the best use I could make of such an opportunity, and finally decided to fall back on my personal experience in London of organic heart disease in childhood.

You must not think that I have only concerned myself with heart disease in childhood, for I have been in charge of adults also at University College Hospital for over thirty years. One of my chief concerns with children has been to investigate the origin and possible prevention of early organic heart disease, and my experience with adults has been a continual incentive to pursue that study.

It is not my intention to weary you with the pathological investigations I made of rheumatic carditis and malignant endocarditis, of diphtheria and septic infections, or with the investigations into the cause of acute rheumatism made with my late friend and great research worker Dr. Alexander Paine. Some of these were made in the last century, and have been elaborated or improved upon or passed over.

I should not, however, be doing due honour to the University College from which I received my early training if I did not claim that these investigations anticipated the Aschoff's nodules, demonstrated the essential damage done to the myocardium by rheumatism, and contributed the first experimental proof in this country of the production of carditis from the active lesions of acute rheumatism.[1,2,3] It is, I think, remarkable that a Bristol physiologist, Dr. Kent, in 1892 demonstrated clearly and decisively what is now called the bundle of His, and that the further work of the late Dr. Carey Coombs, Dr. C. E. K. Herapath, Professor G. Hadfield and Dr. Bruce Perry on the rheumatic heart has again carried on the advance of our knowledge of this subject. Moreover, Bristol has been the pioneer in the provinces by establishing at Winford a school hospital.

I have no doubt that to the teaching at the school of Bristol will in the future be given the credit for much of the pioneer work done in this country on the myocardium, and no one will deny that the myocardium is the main factor in determining the course of most cases of organic heart disease.

### RHEUMATIC HEART DISEASE.

We do not know the nature of the poisons of rheumatism, but clinical experience has taught us that these must differ much in virulence in different children. Thus strong children of about ten years of age may recover so wonderfully from an acute carditis that years later no one could believe that there had been a severe pericarditis. On the other hand, rheumatism may wreck the myocardium, and the autopsy demonstrates that the valvular damage could not have greatly injured the function of the heart, so slight was it in its degree.

It is not sufficiently realized that acute so-called "simple" rheumatic endocarditis is not fatal, so that when death occurs from a general carditis there has been time for the infective micrococci of rheumatism to be destroyed in the vegetations on the valves, or if not destroyed to require the utmost care and patience in their discovery, and the same is true of experimental heart disease. The exception to this is malignant rheumatic endocarditis, which most observers do not recognize, claiming that this form of endocarditis is always the result of a secondary infection, a question to which I will revert later. It is, I also think, only those who have studied the infection in animals and traced it through every stage who can realize how effectually the tissues, cells and leucocytes combine to destroy the strepto-diplococci in the tissues.

However, my intention is not to dwell upon pathology, but upon the more clinical aspect of organic heart disease in childhood as I have seen it in London.

The rheumatic type varies much in frequency and virulence, not only in individual cases but in different years. Last year, in the autumn, after the long, dry summer, the cases were severe, and I never saw subcutaneous nodules so frequently, nor that sub-periosteal inflammation over bony prominences which leads to the "growing out," as I term it, of the bones of the wrists, of the elbows and ankles. In one case a bony node was detected on the shaft of the left femur, such as I have seen produced by experiment from an intravenous inoculation of a rabbit with the strepto-diplococcus. It is many years since Sir Arthur Newsholme insisted that rheumatism was essentially an urban disease, rife when the sub-surface level of water was low. During last autumn there were also extensive outbreaks of tonsillitis in London, and we may be sure that when these outbreaks occur there will be much acute rheumatism among London children as the bright, warm weather swiftly turns to grey cold.

### *Apyrexial Carditis.*

The most deadly form of rheumatic heart disease is the apyrexial type, with a stealthy carditis, nodules and rapid anæmia. I know no remedy for it—salicylates, tolysin, rest and splendid nursing all fail. Fortunately it is not very frequent. Not only may it be deadly, but it is easily underrated when it does occur, and the inexperienced, missing the cardiac dilatation, do not realize the importance of the rapid pulse, and may easily enough overlook a warning subcutaneous nodule. This type chiefly affects the very young, and I should venture to traverse the frequent statement that acute rheumatic heart disease is rare under five years. I have repeatedly seen it occur under that age, and taking a hundred consecutive cases of varying severity in my ward I found 10 per cent. began before the age of five years. Doctors should not be led astray by the belief that this early rheumatism is most exceptional, and must not forget that the mortality at that age is in proportion greater than at any period.

### *Hyperpyrexia.*

This dreadful apyrexial type becomes the more arresting and remarkable when contrasted with the case of a boy of fifteen years who came under my care last year wildly delirious and incontinent, with a temperature of over 106° F., carditis and arthritis. He, in spite of general pericarditis, aortic and mitral disease, has recovered so marvellously that it is with difficulty now that any murmur at all can be detected, and he is back at work, cheerful and strong.

Hyperpyrexia in rheumatism is not a thing of the past, for in addition to this case I have recently seen two cases of chorea with rheumatic arthritis and a temperature of nearly 107° F. These reacted well to cold sponging.

It cannot be too strongly insisted that to look on lowering of the temperature as any proof of the specific efficacy of salicylates in the acute rheumatism of childhood is fallacious, seeing that the most virulent cases are apyrexial; but it is difficult indeed to convince students and even doctors that the intensive—I stress the word "intensive"—treatment of acute carditis in early childhood with salicylates is not only unsuccessful but may be fatal.

### *Venous Thrombosis.*

There is one event in the rheumatic heart disease of childhood that has always interested me, and upon which I published an early investigation in 1898.[4] My interest has been lately refreshed by a Bristol physician, Dr. Bruce Perry, one of my former house-physicians at Great Ormond Street, who independently in this city confirmed these observations.[5] This event is the occurrence of thrombosis of large veins. I published originally three cases, but I have seen ten examples, and have not a shadow of doubt as to its reality, though few other observers in this country have recorded its occurrence. The axillary and innominate veins have in my experience been the most frequently affected, but one of my first cases, which proved fatal, showed thrombosis of the superior vena cava and all its main tributaries, death occurring from a cardiac embolism.

Those who have not seen this condition may object that it is a mechanical result, brought about by a severe pericarditis with adhesions around the large veins entering the heart, aided by the failing circulation. A. F. Voelcker made this explanation unlikely when he described a case of chorea (and chorea in this country as a rule is undoubtedly an active rheumatism) in which, although there was no cardiac disease, there were thromboses in the femoral and saphena veins.[6] Again, in 1927, in a paper with the rather fantastic title "Inspiring Cases of Rheumatism,"[7] I described a case of severe chorea with carditis and venous thrombosis of the inferior vena cava with recovery. There was later the development of that striking collateral circulation such as we sometimes see after the same event in typhoid fever.

Clearly, then, we can put aside once and for all an explanation based on a mechanical interference associated with pericardial adhesions. This was to me evident from my first observations, though these were unfortunately made before I had commenced investigating the streptococcal cause of acute rheumatism. The evidence lay in the fact that the veins in which the thrombosis occurred showed acute inflammation and changes comparable to those so carefully described by Dr. Bruce Perry. These resemble those occasionally seen in the rheumatic arteritis of childhood, of which I have a striking example, made from a section of the aorta of a child who died of virulent rheumatism at the age of three years. Whether or not the phlebitis is due to some remarkable change in the blood or is due to a local infection may be disputed, but I incline to the latter view. The diagnosis is easy, and the more easy because everyone will admit that cardiac œdema is not a striking feature of acute carditis in childhood. Indeed, one of the remarkable features of the œdema in this acute carditis is a slight puffiness of the face, which suggests a renal rather than a cardiac œdema, although there is no evidence of renal disease that one recognizes. If, then, we come to the bedside of one of these cases and find an unmistakable unilateral swelling of an arm and hand the diagnosis is instantly clear, and if the external and internal jugular veins are affected the tender hard cords of the veins in which the thrombosis has occurred will be palpable.

Similarly, if we find both lower extremities rapidly becoming much swollen with œdema we know that unless it is the final stage of congestive failure due to chronic rheumatic valvular disease, in an acute carditis it must be due to a venous thrombosis.

These cases are not all fatal. On the contrary, five I have watched recovered from the thrombosis, though cardiac lesions may be left of varying degrees of severity, and in my experience they are generally severe.

In passing I would mention that in acutely virulent cases of rheumatism you may not find Aschoff's nodules, just as in virulent acute tuberculosis you may not find any giant cell formation.

The earliest focal lesion in virulent rheumatism affecting the heart I consider to be an area of necrosis in the connective tissue, and around this develop the Aschoff nodules, which I interpret as the first stage in the healing of the focal lesion. This I would compare with the subcutaneous nodule, which in the early stages shows on section a central small core of necrosis, a middle area of cellular infiltration, and an outer zone of swollen connective tissue with distended blood capillaries. The central core of necrosis is in the cardiac lesion minute, and as in the subcutaneous nodule the amount of scar formation is very small, provided the rheumatic process is not prolonged and recurrent, when there may result a considerable fibrosis. I distinguish between these focal connective tissue lesions and the general damage to the myocardium by the rheumatic poison.

### *Heart Block.*

Under this heading I will content myself with citing one case which illustrates the intense interest that surrounds early rheumatic heart disease. There is no point about it that has not been recorded by others, but its value is in the completeness of the history.

I saw this patient when a boy aged 13 years, in 1926. He had a sore throat and had fainted several times, had vomited and complained of pain in his chest. He was a nervous, clever, athletic boy who had usually excellent health. When I saw him in bed his throat was inflamed, his pulse 36 to the minute, but the jugular pulsations were 72, and I diagnosed heart block with probable Stokes-Adams symptoms. He was transferred to hospital for investigation, but the heart block disappeared and a slight mitral systolic murmur became audible. This was followed by a formidable attack of acute rheumatism with multiple arthritis, chorea and aortic and mitral valvulitis. He was completely dumb with the chorea and took months to recover from his illness, leaving hospital with a mitral lesion and aortic regurgitation. He then apparently remained well, subsequently passed his B.Sc. of London, and obtained a scientific post of some promise. Before, however, he actually commenced this work he complained of breathlessness, became anæmic, and had arthritic and muscular pains for which he came to see me at the hospital. He had grown well in the interval, was most intelligent, and had never been laid up since his severe illness. The anæmia was obvious, the valvular lesions with the aortic predominant were present, the temperature was raised, and the heart excited in action. On the tips of his fingers were Osler's spots, which he had himself noticed. The diagnosis of acute malignant endocarditis was only too obvious, and shortly afterwards a large cerebral embolism occurred, followed by death.

Here we have this sequence: the well-known but comparatively rare commencement of acute rheumatism with heart block following a sore throat, a fulminating acute attack of the disease, a period of quiescence and then, without any obvious cause, the final tragedy of fatal acute malignant endocarditis. This is the only case I have traced from an early passing heart block to death, although we know that death may follow from a general carditis in such cases, but I have traced many examples of rheumatic aortic and mitral disease to death from malignant endocarditis, to which I now turn.

### *Malignant Endocarditis.*

There are few more vitally important and interesting problems concerned with rheumatism than that of malignant endocarditis, and there is not one of us who has not felt the utter hopelessness and futility of our efforts to arrest its progress.

We know there are many causes of malignant endocarditis, and we know, too, that it is comparatively infrequent in childhood; we know also that in the rheumatic it is generally looked upon as a secondary infection (usually by streptococci) of a damaged valve or valves. The mention of streptococci at once makes us think carefully, particularly if we admit that a streptococcus is a cause of acute rheumatism.

Again, we have heard and read much of the streptococcus viridans and hæmolyticus as studied in the bacteriologist's laboratory, but for a few moments I would venture to direct attention to a clinical side of acute rheumatism.

Many years ago, in 1899, I published a case of very extensive purpura during an attack of acute rheumatism,[8] which at autopsy showed the lesions that are accepted as those of this disease and from which during life Dr. A. Paine isolated a streptococcus. From time to time since I have seen considerable purpura in acute rheumatism, and have also seen rheumatic nodules surrounded by zones of purpura. The most frequent cause of a hæmorrhagic pericardial exudation in childhood is acute rheumatism. Epistaxis is a well-known symptom, and I personally believe a hæmorrhagic nephritis may occasionally be rheumatic ; and Dr. Paine experimentally produced a hæmorrhagic nephritis with the strepto-diplococcus.

It is clear to me, then, from clinical study that if rheumatism is streptococcal in origin the streptococcus must be certainly at times hæmolytic. On the other hand, not all cases of rheumatism in childhood show hæmorrhagic symptoms, yet develop the classical lesions, and thus it appears to me also that there is no cardinal distinction to be made in clinical medicine between a streptococcus which is hæmolytic and non-hæmolytic if this be expressed in terms of human disease. I would interpret this as meaning that there are factors, not yet understood, dependent not only on the streptococcus, but the individual attacked, which govern the phenomenon of hæmolysis. In other words, I am not yet convinced that we can judge the exact rôle in human disease taken by the streptococcus when it is once isolated from the human tissues and studied *in vitro*.

The point of this digression is this : that it is possible to me that a streptococcus lurking in the system may change its characters when those tissues are altered by general ill-health or bad surroundings, or even by long sojourn in human tissues, and that the rigid view of a secondary infection to account for malignant endocarditis is not to be accepted.

If it is accepted, why is malignant endocarditis uncommon in childhood, the age at which acute rheumatism is rife ? Have not children repeated occasions for secondary infections ? Do they not, many of them, live in stuffy rooms, and are not many under-nourished ? Why, then, is malignant endocarditis infrequent in childhood, seeing that many rheumatic children have sores or otitis or mastoiditis or nasal suppurations ? Rheumatic children in my opinion are relatively more often exposed to secondary infection than the adult, and for me this rigid view of secondary infection palpably fails to explain the clinical fact of the comparative rarity of malignant endocarditis in the child.

Turning to microscopy, what do we sometimes find when we carefully and persistently examine many sections of a rheumatic endocarditis ? We find in acute cases the streptococci in what I call the "fighting area," that is to say, where the area of necrosis in a vegetation meets the live part of the valve in which there is active leucocytosis and tissue reaction. These are scanty, they may be scanty even in experimental endocarditis (which is a crude and gross infection), for the organism is rapidly destroyed by the tissues, or I would rather say destroyed so far as our staining methods can show. Again, we may find in chronic cases in the human being small areas of necrosis in a rheumatic valve shut off more or less imperfectly by fibrous tissue.

Two questions arise. First, are we certain when a rheumatic endocarditis has completely healed and, if so, how are we certain ? Secondly, are we sure that the streptococci, though not visible in the necrotic area of a rheumatic valve, are certainly destroyed or only that they lose their power to retain stains ? In fact, are "destruction" and "loss of staining power" interchangeable terms ? Again, the streptococci may be lurking in the immediate neighbourhood, not in the necrotic tissue but in the living valve tissue itself.

In order to emphasize the importance of these questions, I must turn for a moment from actual childhood to a young medical student who died of malignant endocarditis. He had, when at hospital, an acute rheumatic attack, which left him with severe aortic regurgitation and a mitral lesion. Later, after leaving hospital, he would come to see me at intervals whenever he had warnings of fresh rheumatism. He was clever and became a most promising doctor. Every time he came to see me he said: "I believe I have malignant endocarditis," and every time I laughed away his fears, which were also mine. He later settled down to work for about two years free of definite rheumatism, but with severe valvular lesions, a very large heart and occasional arthritic pains. At last, without any obvious reason—and how often, I ask, is the reason obvious?—he developed more rheumatic pains, and became very tired and breathless, and there was some fever. Streptococci were found in the blood, and the fear had now become a certainty. He had malignant endocarditis. While under observation during that final illness he developed an acute arthritis affecting his knees, wrists and hands. This gave the poor fellow great hope, for we had not let him know the truth we feared, and in that development he saw a return of his old enemy, acute rheumatism, which I believe it was. This arthritis with effusion subsided completely under treatment, but he died of the malignant endocarditis.

I feel morally certain that through the five years after his first attack of acute rheumatism those cardiac valves had never really healed, and I do not believe there was a secondary, non-rheumatic infection.

In children I have seen rheumatic pericarditis subside and multiple arthritis vanish, and yet death occur in the same illness from malignant endocarditis. Are we to assume two simultaneous infections with two different streptococci, one only attacking the valves of the heart, the other the pericardium and joints?

I have cut rheumatic nodules and demonstrated streptococci in diplococcal form in the "fighting zone."

I have cut a large subcutaneous nodule over the elbow in malignant endocarditis, and demonstrated streptococci in diplococcal form in the "fighting area," and in a blood capillary just external to that area.

I have shown at the Medical Society of London the heart[9] of a child who had died from severe carditis with multiple arthritis. The vegetations on the aortic cusps were classically rheumatic, and they were the same on the mitral valve except for one, and this was a large fungating vegetation which was a classical example of acute malignant endocarditis. Was this vegetation singled out by some secondary or superimposed infection, and all the other lesions left to the rheumatic infection ?

A severe aortic and mitral lesion developing in the same attack of rheumatism always suggests to me a warning of malignancy, and my experience supports my fear, for I have collected a number of these cases which died eventually of this form of endocarditis.[10] Within three months in 1932 I had four such cases in young people with this double lesion dating from childhood. One of them, a young ward sister, developed multiple painful arthritis with effusion reacting to salicylates. It was an illness precisely resembling acute rheumatism, but her temperature remained raised, and looking at her finger-tips I saw the fatal Osler's spots.

In some respects the most remarkable case of organic heart disease in childhood met with in my experience was the following. The mother of an infant had an attack of acute rheumatism during late pregnancy from which she recovered, but her child was born deeply cyanosed, and died on the third day. The post-mortem examination showed an extreme endocarditis of the mitral valve with fungating vegetations which contained numerous streptococci. There was no evidence of a suppurative lesion at the umbilicus or elsewhere, and even if this had been found the fact that the child was born deeply cyanosed and had no congenital malformation of the heart would have been difficult to interpret in any way other than as the result of an ante-natal infection.

These, then, are some of the reasons upon which I base my view that there is a rheumatic malignant endocarditis. The explanation is not a complete one: it cannot be with the present uncertainty of medical thought, but I seem to see some further light dawning in favour of the view. For many years I have believed that the rheumatic poisons were so subtle that they built themselves into the living cells of the body. This is, I admit, a primitive thought, but somewhat supported by the recent studies of allergy. This term I use with the utmost caution, and say at once that when a child develops a first attack of acute rheumatism with general carditis that is not in my opinion an allergic condition. Acute rheumatism is, I hold, not allergic, though I believe it to have in its life history an element of allergy, and that the body when once attacked by acute rheumatism may become sensitized to that streptococcal infection. Then may result a failure of the tissue cells and leucocytes in a damaged cardiac valve to destroy lurking streptococci, and a failure of the entire system to react against the fatal focus lying directly in the track of the blood-stream. There is no other rheumatic lesion that lies in such a relation to the circulation, but I believe a pericarditis may also be malignant in type, though by its anatomical arrangement it cannot produce a condition comparable to that of a malignant endocarditis. The problem of malignant endocarditis in the rheumatic is, I hold, not to be solved by an easy assumption of a super-imposed infection, but requires a more prolonged study of rheumatic endocarditis, of the disease "rheumatism," and also of the streptococcus.

In touching upon allergy I do no more than show that I am alive to that aspect, and I should also wish you to know that I have also in mind the recent work of Professor Hadfield and his colleagues[13] upon the lysis of fibrin by hæmolytic streptococci, which is of deep interest.

### *A Transitional Type of Rheumatic Endocarditis.*

In January, 1934, a boy of six years illustrated another feature in this problem. This boy was admitted with pericarditis, mediastinitis, endocarditis of the mitral and aortic valves, and numerous subcutaneous nodules. It was not his first attack of rheumatism, and the physical signs pointed to an adherent pericardium. The acute attack died down and he became a very cheery boy, yet in spite of this began to lose ground slowly, and though for weeks before his death there was no fever, he wasted, his pulse remained persistently rapid, and his face became pinched and livid. In this toxic condition he died. From previous experience I demonstrated upon him as a malignant rheumatic endocarditis of the transitional type.

The autopsy showed the adherent pericardium and mediastinitis. The aortic and mitral valves showed vegetations, those on the aortic of the usual rheumatic type, those on the mitral larger, and resembling those small vegetations you see in some areas of a frank malignant endocarditis or on the mural aspect of the endocardium.

Such cases Dr. Paine and I described in 1912,[11] and republished in "Researches on Rheumatism." I think it is important that the diagnosis was made in this case during life and confirmed after death, and such a case provides, in my opinion, a missing link in our problem which may be easily overlooked. There is the clinical "simple" rheumatic endocarditis, there is the malignant type occurring in a rheumatic subject, and joining these a rheumatic type malignant in its toxicity and persistence, but with vegetations which if looked upon as a case of acute rheumatism would be called large, or if looked upon as a case of malignant endocarditis would be called small. I have dwelt so long upon this subject because I feel there is some hope that, if my view is correct, we may eventually prevent the rheumatic form of malignant endocarditis, and at least this is a line of approach less hopeless than the present one of negativism. It has received of late increasing support upon the Continent, and I need only mention in proof of this the names of Professors Talaliev, Schotmüller, Herschfield, Lang, Orth and Hasincamf.

### TUBERCULOUS HEART DISEASE.

Tuberculous heart disease has been in my experience uncommon among children in London. I put aside general miliary tuberculosis, for the cardiac lesions are so minute as to be of no practical importance as compared with the pulmonary and meningeal lesions. I have, however, seen sufficient of this heart disease to get a clear idea of the general clinical features in the child. Tuberculous pericarditis is the lesion most commonly met with, but valvular heart disease may occur with it, or independently of it, though I believe myself correct in stating valvular lesions are rare, and these when chronic may show much calcification, as I have seen. It is very probable that we shall mistake the early signs of tuberculous pericarditis for rheumatic pericarditis, because the rheumatic form is so much the more frequent. Nevertheless, I think we may be suspicious when there are no other signs of rheumatism, although we must admit that a primary cardiac rheumatism—

S

that is, a rheumatism of the heart with no arthritic pains, no joint swellings and no chorea—may certainly occur and be severe enough even to prove fatal. Again, I think we may be also suspicious if there is pericarditis without detectable valvular disease, for this in rheumatism is unusual, but though it is unusual this again certainly occurs clinically, and on the other hand, as already mentioned, tuberculous endocarditis is sometimes present. Again, the Mantoux test may help us in a suspicious case.

The tuberculous form of pericarditis takes two different clinical courses, with the usual reservation of the clinician that there may be transitional cases. In the first place it may cause a large serous or blood-stained effusion for which paracentesis of the pericardium may have to be undertaken not once only but sometimes twice or thrice. In this effusion the tubercle bacilli may be found in some cases in great numbers, but in others they cannot be found, and the diagnosis is confirmed perhaps by the infection of a guinea-pig, although we may be morally certain from the character of the fluid and the excess of lymphocytes as to its nature.

Paracentesis of the pericardium for a child under twelve years of age on account of rheumatic pericarditis is so rarely indicated in London that I have not had a case since the War. In spite, then, of difficulties we generally arrive at the correct diagnosis of the nature of tuberculous pericarditis when we are driven to paracentesis. Such cases may die from the acute condition or recover, possibly to die later from a general tuberculosis.

Very different and very remarkable is the plastic type of tuberculous pericarditis. I have had opportunities of following such cases from the commencement to the end.

The pericarditis is a part of a general subacute tuberculosis of the serous membranes, and may be the first membrane attacked or appear later when a tuberculous pleurisy has been detected and subsided under treatment.

When it is the first event there is the usual difficulty of excluding rheumatic pericarditis upon which I have already dwelt. The pericardial friction is indistinguishable, but there is not the cardiac dilatation met with in rheumatism, and as there is probably no valvular disease you feel at the back of your mind that the case is an unusual one, but may get no farther than that. Slowly the pericarditis subsides after some weeks of irregular fever, and the child may leave the hospital apparently cured, or recovery may be interrupted by a pleurisy on the left side, or either pleura may be singly affected. There is usually no effusion with this pleurisy, but the subsidence is slow and the signs of a thickened pleura follow. Eventually the child partially recovers, and a year or two later, during which time the health has been indifferent, is brought to hospital for ascites. It may happen that the early stage I have described may not have been under observation, and then we are confronted by a child with a mysterious ascites. The immediate diagnosis is generally tuberculous peritonitis, but this is unsettled by the much enlarged liver and spleen and the insistence of signs of cardiac weakness.

Examination of the heart requires minute care, for the solution of the problem lies there. The cardiac dullness may be but little increased, but in a classical case it seems to be unusually massive and wooden, and on listening to the heart the sounds are muffled and feeble, particularly the first sound. Again, there will probably be no valvular murmur. Examination of the chest may discover a diminution of the air entry in one or both axillæ and a loss of resonance on percussion, and a sense of resistance which suggests a thickened pleura.

The pathology of the condition explains the cardiac signs. The pericardium is greatly thickened with possibly calcareous plaques embedded in it, and the heart is of small size and strangled by the thick pericardium. The ascites gains ground, and many repeated paracenteses of the abdomen are needed. In this pitiable state the child may survive for a year or more, and then die from exhaustion, or there may be the involvement of another serous membrane which may give rise to symptoms that are liable to be most puzzling if the case is not understood. Ptosis may develop with headache, retraction of the neck, vomiting and convulsions. The serous membrane of the brain has at last been attacked, and death speedily follows. At the post - mortem examination the peritoneum, though possibly thickened from prolonged ascites, may not show any tubercle. Not all cases die from meningitis, but the above is the classical course which I have followed with care through every phase in three cases. Only once have I seen a multiple serositis with repeated ascites in the acute rheumatism of childhood. In this case there was an adherent pericardium and chronic rheumatic peritonitis, in the early phase of which I detected peritoneal friction over the liver and spleen. This child I followed for more than two years, and an attempt to deal with the ascites surgically proved fatal.

A case of tuberculous pericarditis of this type[12] was the occasion for the first example of Brauer's operation for cardiolysis published in this country, though Allingham had already undertaken this procedure. In my case the patient was a boy of 13, who even when he walked about the hospital ward developed œdema of the legs and breathlessness. There was no valvular disease, but massive precordial dullness and remarkable diastolic recession of the intercostal spaces on the left side over a wide area. I had thought the case was rheumatic. Mr. Trotter, on opening the chest, found a large tough adhesion shackling to the parietes the area around the heart's impulse. It was a very remarkable sight to see the heart spring back when this was severed.

Recovery was remarkable, and for two summers the boy was well enough to act as a scorer for his school cricket club. Then he developed a left pleurisy with effusion and this was tuberculous. Later he died of pulmonary tuberculosis, but no autopsy was allowed.

I have never advised this operation for a rheumatic child, either because I was uncertain of my diagnosis or because there was in my opinion too extensive valvular disease, or perhaps because I was not sufficiently courageous. Others, however, have reported successes.

### PNEUMOCOCCAL HEART DISEASE.

In London pneumococcal heart disease in childhood is in my opinion infrequent, though it cannot be called rare, and this infection usually attacks the pericardium. I can only recall one case of acute pneumococcal endocarditis in an infant. This child of eighteen months had an acute apical pneumonia with high fever, and developed a loud aortic systolic murmur. After death there was found a large fungating vegetation on one of the aortic cusps which gave a pure culture of the pneumococcus lanceolatus.

Pneumococcal pericarditis, which we know is more frequent in children of under four years than at any other age in childhood, may occur at all ages.

Undoubtedly this pericarditis may spread direct from a pneumococcal pleurisy, and in former days, when empyemata were not dealt with surgically, the end was not uncommonly suppurative pericarditis. It is, however, generally an independent infection of the pericardium, and this was strikingly illustrated in one of my cases in which the left upper lobe was solid with pneumonia, but neither its pleura abutting on the heart nor the corresponding external surface of the pericardium was implicated, although there was extensive internal pneumococcal pericarditis. This form may, we know, be accompanied by a copious purulent effusion, and from time to time we see and read of cases which have been successfully operated upon. In some of the cases with great effusion I have known the heart sounds completely disappear. The prognosis is always serious.

I would again venture to dwell upon this point, the rarity of malignant endocarditis in the pneumococcal infections of childhood.

Very different are the more frequent cases in early childhood, and I think the diagnosis is one of the most difficult in heart disease. Often enough, it is true, the diagnosis is of no practical importance, for opening the pericardium could be of no service when, after death, you find only a thin plaster of pus, and the child has evidently died from a severe pneumonia with possibly empyema, peritonitis and meningitis. Every clinician is, however, disturbed when he finds a lesion at autopsy which he has not even suspected during life, and asks himself the reasons for the failure. One reason, and a formidable one, is the usual absence of pericardial friction. It is very interesting to realize the difficulty there is in the diagnosis of pericarditis when there is no pericardial friction, particularly when there are also no endocarditis, and no great dilatation of the heart, as in rheumatism, to attract your attention. I have some interesting figures upon these points.

From a study of 100 fatal examples in childhood I found that about 80 per cent. of cases of pyo-pericardium occur under four years, and about 60 per cent. at three years and under. There was no practical difference in the number of cases that were associated with an empyema on the left side as compared with the right or with empyemata on both sides. A large purulent effusion was exceptional, and in only about 25 per cent. did the amount of pus found in the pericardium exceed three ounces.

At one time I had in my mind that pneumococcal pericarditis was rapidly fatal, but from my study of these cases I was disabused of this view, for in seventeen there was evidence of a much thickened pericardium and of much attempt at organization of the exudation, changes which must have taken months to have developed. I only found a record of pericardial friction in four cases, but this cannot be accepted as more than a proof of its infrequence.

Then, again, another difficulty in the diagnosis of this form of pericarditis is that the child is generally very ill with respiratory lesions, and when the temperature remains high or irregular we naturally search for an empyema, and sometimes when completely puzzled fall into the trap of changing the diagnosis to one of acute tuberculosis.

Fainting attacks, rapid and changing rates of pulse and irregular spikes of fever have been observed in these cases, but I have not found them at all constant, and certainly fainting attacks may not occur. Another difficulty is one which is irritating. You see a case, and vow that you will not miss the next one; but the interval of time before you see the next case is just so long that you forget your vow and fail again. Fortunately, as I have said, failure may be of little practical importance, and those cases which are urgent, those with a considerable effusion, give those clinical signs that are recorded in every text-book and radiography also comes to our assistance.

I end this lecture in the spirit of Research—a research to link the emotional with the physical heart. Physicians of experience have frequently noticed that when a case of organic heart disease—a case, for example, of a hard-working artisan—is nearing the end, and I have noticed this particularly with cases of aortic regurgitation, there comes upon him a curious state in which, however ill, he insists upon going home, and his faithful wife, in spite of anxiety and danger, welcomes him back.

Some here will know what it means to work year in year out at a subject until Time touches him on the shoulder. Those will then understand what pleasure it gave me when I received from this University—which casts its influence over a part of the country my family have been connected with for over 150 years—the invitation to give this lecture, a welcome to the lad of seventeen to come back and tell them some of his work and experiences. This invitation has greatly pleased me, for it is the first greeting I have received from our own universities, though not the first from our Dominions and America. I have, then, asked your Faculty of Medicine to accept as a thank-offering the original manuscripts of the two papers which I have thought to be the most important that I wrote with my late colleague Dr. A. Paine on the subject of acute rheumatism, and with them our collected papers as published in "Researches on Rheumatism."

These are now naturally documents of the past, but it is possible that in future days these manuscripts may be of interest to the medical historian and to this University as the work of two Somersetshire men.

### REFERENCES.

[1] Coombs, Carey F., *Rheumatic Heart Disease.* John Wright & Sons Ltd. 1924.

[2] Poynton and Paine, "Researches on Rheumatism," Paper No. 2, *Quart. Jour. of Med.*, 1899.

[3] Poynton and Paine, "Researches on Rheumatism," Paper No. 8, *Quart. Jour. of Med.*, 1900.

[4] Poynton and Paine, "Researches on Rheumatism," Paper No. 5, *Quart. Jour. of Med.*, 1898.

[5] Perry, C. B., "Thrombo-phlebitis in Acute Rheumatism," *Lancet*, 28th October, 1933, p. 966.

[6] Voelcker, A. F., *Diseases of Children.* Garrod, Batten and Thursfield. Edward Arnold & Co. 1913.

[7] Poynton, F. J., "Some Inspiring Cases of Rheumatism," *Archives of Disease in Childhood*, February, 1927.

[8] Poynton, F. J., "Case of Virulent Acute Rheumatism with Extensive Purpura," *Lancet*, 8th October, 1899.

[9] Poynton, F. J., *Trans. Med. Soc.* Lond., 1911, xxxiv. 230.

[10] Poynton and Paine, "Researches on Rheumatism," Paper No. 15, *Medico-Chir. Trans.*, vol. lxxxv.

[11] Poynton and Paine, "Researches on Rheumatism," Paper No. 23, *Quart. Jour. of Med.*, July, 1912.

[12] Poynton and Trotter, "On the Operation of Cardiolysis," *Lancet*, 1909, i. 1,742.

[13] Hadfield, Magee and Perry, "The Lysis of Fibrin by Streptococci," *Lancet*, 1934, i. 834.

